# Effect of partial or total supplementation of soybean meal with fluted pumpkin (*Telfairia occidentalis*) seed meal in the diet of hybrid Catfish *(Heteroclarias)* fingerlings

**DOI:** 10.1002/fsn3.659

**Published:** 2018-05-02

**Authors:** Olamiposi Olajumoke Omosowone, Abibat Motunrayo Ogunrinde

**Affiliations:** ^1^ Department of Fisheries and Aquaculture Technology Federal University of Technology Akure Akure Ondo State Nigeria

**Keywords:** catfish, fluted pumpkin, partial or total supplementation, soybean

## Abstract

The study evaluated the effect of partial or total supplementation of soybean meal with full‐fat *Telifaria occidentalis* seed meal on performance and whole body composition of *Heteroclarias* fingerlings. Five iso‐nitrogenous (40% crude protein) basal diets were prepared using locally available feedstuffs, while *T. occidentalis* seed meal was added at varying inclusion levels (0%, 25%, 50%, 75% and 100%). One hundred and fifty apparently healthy *Heteroclarias* fingerlings (4.26 ± 0.26 g) were stocked in glass tanks (50 L capacity) at 10 fish / tank with each treatment in triplicate. Fish were fed at 5% body weight in two equal portions daily for 56 days and culture water changed on alternate days. The result of this study showed a significant variation (*p *< .05) in crude protein and lipid contents of whole body composition of *Heteroclarias* fingerlings‐fed *T. occidentalis* seed meal supplemented diets compared with the control. Increasing levels of fat and protein deposition were also observed in the muscle of *Heteroclarias* with increase in *T. occidentalis*. Also, some growth evaluation indices (weight gain, specific growth rate, feed intake, and protein efficiency ratio) of *Heteroclarias* fingerlings decreased with increase in inclusion levels of *T. occidentalis* seed meal. Dissolved oxygen in culture units also decreased with increase in *T. occidentalis*. From this study, chemical body composition of *Heteroclarias* fed *T. occidentalis* seed meal supplemented diets increased with increase in inclusion levels, while the reverse was the case in weight gain. There was a decline in the quantity of dissolved oxygen with increase in quantity of *T. occidentalis* seed meal. Other water parameters such as pH, temperature, nitrate, and ammonia were within the limits for culturing tropical fish species.

## 1 INTRODUCTION

Fish is a high‐quality food containing first‐class protein and nutrients, important for human health and growth (Olaifa, Oladapo, & Bello, 2010). Different fish species have been used as foods by humans from ancient times and form an important part of the diet in many countries (Haard, 1992 and Macrae, Robinson, & Sadler, 1993). They are generally low in cholesterol compared to meat from terrestrial animals because they contain a wide range of lipids high in polyunsaturated fatty acids (Bell, 1988). The expansion of aquaculture and increase in livestock production strongly indicates that a “feed crises” will be inevitable in the nearest future if possible solutions are not proffered. Increasing competition in the feed industry plus rising cost of traditional feed ingredients in recent years are forcing feed manufacturers to find alternatives to conventional feed stuffs and cost of feeding constitutes 60% of the total production cost. There are numerous raw materials of plant origin with low cost when compared to that of animal origin, as such, are potential replacements to the former. Cereal and oilseed proteins play prominent roles in the diets of omnivorous fish species such as catfishes, carps, and tilapias (Naylor et al., 2009). The proportion of fishmeal and alternative protein ingredients in aquafeeds depends on the nutritional requirements of the species, stage of development, and the regulatory environment of production systems. Feed stuffs of plant origin often possess high‐binding capacity which is associated with the presence of digestible or indigestible substances (Pike & Hardy, 1997). The major limitations associated with plant feedstuffs is the presence of antinutrients and less palatability. *Telfairia occidentalis* (fluted pumpkin) seeds have high nutritive values such as protein, fats and oils, and minerals (potassium, iron) but their utilization could be affected by the presence of antinutritional factors such as lectins and trypsin inhibitor that are present in the seeds (Kuku, Etti, & Ibironke, 2014). Several researchers at different times have evaluated the importance of various parts of the plant. The plant is important for its edible seeds which are rich in protein and fat (Ajiboye, Yakubu, & Adams, 2012); the leaves constitute an important component of the diet of many people in West African Countries (Akwaowo, Ndon, & Etuk, 2000) and are rich sources of protein, oil, vitamins, minerals, which nourishes, protects and heals the body (Nwanna, Falaye, & Sotolu, 2008). The young shoot and leaves of the plants are also used as soup because of the pleasant taste (Odiaka, Akoroda, & Odiaka, 2008) and due to the richness of the leaves in iron, it is used to cure anemia (Ajibade, Balogun, Afolabi, & Kupolati, 2006). The seeds are valuable as a high protein for human and animal food and used in herbal preparation for the treatment of sudden attack of convulsion, malaria, and anemia (Ladeji, Okoye, & Ojobe, 1995). Growth of fish depends largely on good nutrition which is more pronounced with fish raised in intensive aquaculture system. In fish culture, supplementary feeding plays major roles in determining the nutritional and economic success of the aquaculture venture. Hybrid catfish (*Heteroclarias*) is a good experimental fish in aquaculture due to its relative fast growth rate, better feed conversion efficiency, resistance to diseases, ability to tolerate harsh environmental conditions, and wide acceptance by the populace (Aluko, 1998 and Omoruwou & Edema, 2011). The objective of this study was to evaluate the effect of partial or total replacement of soybean with *T. occidentalis* seed meal on the growth and whole body composition of *Heteroclarias*.

## 2 MATERIALS AND METHODS

The experiment was carried out in the teaching and research farm of the Department of Fisheries and Aquaculture Technology, The Federal University of Technology, Akure. Five iso‐nitrogenous (40% crude protein) diets were prepared using same basal conventional feedstuffs. *Telifaria occidentalis* seeds were obtained from local farmers and authenticated by the Department of Crop, Soil and Pest Management of the University. The fluted pumpkin gourds were dissected to remove the coated seed pods, and the pods were broken to expel the seeds. Seeds were oven‐dried to a constant weight at 60°C for 120 hr before grinding using a kitchen blender. Basal ingredients were weighed using a top load balance (Metler Toledo, PB 8001 London), milled separately, and later mixed in different proportions (Table 1). *Telifaria occidentalis* seed meal was added to each batch of the basal ingredients in different proportions (0, 25, 50, 75 and 100) %. All feedstuffs were thoroughly mixed and pelleted using a Hobart A‐2007 pelletizer machine (Hobart Ltd, London, UK) with a 2 mm opening die. Resultant pellets were oven‐dried at 60°C for 24 hr and later refrigerated in airtight bags prior to use. Experimental diets were designated as TOSM1 (control), TOSM2, TOSM3, TOSM4, and TOSM5. Proximate analysis of experimental diets, *T. occidentalis,* and fish followed AOAC 2000 methods (Tables 1, 2 and 3).

**Table 1:** Gross composition (%) of dietary ingredients

Dietary ingredients (%)	TOSM1	TOSM2	TOSM3	TOSM4	TOSM5
Fish meal	27.0	27.0	27.0	27.0	27.0
Soybean meal	27.0	18.0	13.5	9.0	0.0
Yellow maize	36.0	36.0	36.0	36.0	36.0
*Telifaria occidentalis*	0.0	9.0	13.5	18.0	27.0
Vegetable oil	3.0	3.0	3.0	3.0	3.0
Vit./Min. premixes	4.0	4.0	4.0	4.0	4.0
Starch (binder)	3.0	3.0	3.0	3.0	3.0
Proximate composition (dry matter) of experimental diets (%)					
Crude protein	40.25	40.65	41.05	42.87	43.02
Lipid	16.00	17.00	18.02	18.73	19.05
Ash	11.65	12.00	12.03	12.26	12.41
Crude fiber	8.50	6.20	5.20	4.20	3.00
NFE	20.00	20.15	20.06	18.34	19.32
Gross energy (k/cal)^a^	492.61	495.72	503.26	509.38	512.36

a Gross energy: 5.65, 9.45, 4.0 and 4.0 kcal/g of protein, ether extract, crude fiber and NFE (Jobling, 1983).

**Table 2:** Proximate composition (dry matter) of full‐fat *Telifaria Occidentalis* seed meal

Parameter	*T. occidentalis* (%)
Crude ash	4.00
Crude protein	25.20
Crude fiber	12.50
Ether extract	36.00
Nitrogen free extract	15.90

**Table 3:** Whole body composition of *Heteroclarias*‐fed full‐fat *Telifaria occidentalis* seed meal as partial or total replacement for soya bean meal

Parameters (%)	TOSM1	TOSM2	TOSM3	TOSM4	TOSM5
Moisture	7.77 ± 0.06^c^	5.50 ± 0.29^b^	4.00 ± 0.39^a^	5.84 ± 0.10^b^	5.99 ± 0.18^b^
Ash	12.50 ± 0.29^a^	16.50 ± 0.21^bc^	14.50 ± 2.02^b^	16.50 ± 0.87^bc^	17.00 ± 1.15^c^
Crude protein	60.56 ± 1.46^b^	63.91 ± 1.39^b^	67.51 ± 1.33^b^	66.40 ± 0.89^b^	63.50 ± 0.77^a^
Lipid	7.00 ± 0.58^a^	7.25 ± 0.29^b^	7.84 ± 0.29^bc^	8.03 ± 0.29^c^	8.61 ± 0.29^d^
NFE	12.17 ± 0.65^c^	6.84 ± 0.68^b^	6.15 ± 0.79^b^	3.23 ± 0.16^a^	1.01 ± 0.49^a^

Means with the same superscript in the same row are not significantly different (*p *> .05).

One hundred and fifty (150) apparently healthy *Heteroclarias* fingerlings (4.26 ± 0.04) g were acclimatized for 7 days in a holding tank and feed with a farm‐made diet prior to the commencement of feeding trials. Fingerlings were randomly allotted at 10 fingerlings / tank in five dietary groups with each treatment in triplicate. Fish were fed at 5% body weight in two equal rations daily between 8:00–09:00 hr and 16:00–17:00 hr. Rations were adjusted biweekly based on the sampled total weight of the fish in each experimental unit. Some physiochemical water parameters (dissolved oxygen, temperature, pH, nitrate, and ammonia) were also measured (Table 4). At the end of the feeding trials, growth performance was evaluated (Table 5) using indices such as:

**Table 4:** Physiochemical parameters of water in experimental units

Parameters	TOSM1	TOSM2	TOSM3	TOSM4	TOSM5
Dissolved oxygen (mg/L)	5.75 ± 0.26^b^	5.05 ± 0.87^b^	3.65 ± 0.49^a^	3.50 ± 0.12^a^	3.50 ± 0.12^a^
Temperature (°C)	27.50 ± 0.43^a^	26.80 ± 0.12^a^	27.10 ± 0.12^a^	27.5 ± 0.23^a^	27.10 ± 0.12^a^
pH	7.25 ± 0.23^a^	7.20 ± 0.00^a^	7.27 ± 0.01^a^	7.34 ± 0.08^a^	7.22 ± 0.02^a^
Nitrate (mg/L)	0.11 ± 0.01^a^	0.13 ± 0.01^a^	0.12 ± 0.01^a^	0.12 ± 0.01^a^	0.13 ± 0.01^a^
Ammonia (mg/L)	0.002 ± 0.00^a^	0.002 ± 0.00^a^	0.002 ± 0.00^a^	0.003 ± 0.00^a^	0.003 ± 0.00^a^

Means with the same superscript in the same row are not significantly different (*p *> .05).

**Table 5:** Growth evaluation in *Heteroclarias* fed *Telifaria Occidentalis* seed meal as partial or total replacement for soybean meal

Parameters	TOSM1	TOSM2	TOSM3	TOSM4	TOSM5
Initial weight (g)	4.45 ± 0.01^c^	4.03 ± 0.01^a^	4.22 ± 0.08^b^	4.45 ± 0.05^c^	4.17 ± 0.04^ab^
Final weight (g)	25.89 ± 0.25^e^	23.30 ± 3.22^d^	17.77 ± 0.08^c^	15.43 ± 0.10^b^	13.87 ± 0.09^a^
Mean weight gain (g)	21.44 ± 0.25^c^	19.27 ± 3.23^bc^	13.55 ± 0.00^b^	10.98 ± 0.05^a^	9.71 ± 0.05^a^
SGR (%)	1.37 ± 0.03^c^	1.36 ± 0.46^c^	1.12 ± 0.01^b^	0.96 ± 0.01^a^	0.93 ± 0.02^a^
FCR	3.38 ± 0.05^a^	3.39 ± 0.73^a^	3.64 ± 0.32^b^	3.93 ± 0.83^c^	4.00 ± 1.58^d^
PER (%)	0.74 ± 0.06^b^	0.74 ± 0.57^b^	0.68 ± 0.01^ab^	0.63 ± 0.02^a^	0.62 ± 0.03^a^
Survival (%)	100.00 ± 0.00^d^	80.00 ± 1.55^b^	77.00 ± 1.32^b^	90.00 ± 0.00^c^	70.00 ± 0.00^a^
Feed intake (g/day)	3.68 ± 0.08^c^	2.61 ± 1.73^b^	2.49 ± 0.49^b^	1.60 ± 0.13^a^	1.30 ± 0.24^a^

SGR, Specific growth rate; FCR, Feed conversion ratio; PER, Protein efficiency ratio.

Means with the same superscript in the same row are not significantly different (*p *> .05).

Mean weight gain (MWG) = final mean weight − initial mean weight

$$\text{Specific growth rate (SGR)}=\frac{\text{(ln W2)}-\text{(ln W1)}}{T}\times 100$$

where, W1 and W2 are natural log of initial and final weight, and *T* is number of experimental days

$$\text{Protein efficiency ratio (PER)}=\frac{\text{Fish weight gain}}{\text{Protein fed}}$$

where protein fed = % protein × total diet consumed

$$\text{Feed conversion ratio (FCR)}=\frac{\text{Total feed consumed}}{\text{Weight gained}}$$

Feed intake was calculated as given by Falaye and Oloruntuyi (1998)

$$\text{Feed intake (FI)}=\frac{5\%\text{body weight}\times \text{experimental period}}{\text{Number of fish stocked}}$$

All experimental data generated were subjected to one‐way analysis of variance test (ANOVA) using statistical package for social sciences (SPSS) package 22.0, and significant mean differences were separated at 0.05 probability levels according to (Steel, Torrie, & Dickey, 1997).

## 3 RESULTS AND DISCUSSION

Result showed that seeds of full‐fat *T. Occidentalis* had high protein (25.20%) and fat (36.00%) contents (Table 2). Previous researchers have shown the nutritional value of *T. Occidentalis* leaves (Dada, 2015 and Odiaka et al., 2008). Composition of experimental diets revealed crude protein of diets was iso‐nitrogenous between 40.25 and 43.02% (Table 1), therefore, no bias was introduced as a result of nutritional imbalance. Nutritional studies recommended 40–42% crude protein for culture of tropical fishes such as African catfish (Jauncey 1982, Machiels & Henken, 1985 and Uys, 1989). Proximate analyses and bomb calorimetry provide information about the chemical compositions and gross energy contents of feeds but digestibility studies are required to reveal the availabilities of the various nutrients to fish (Friedman, 1996). Wilson (1989) estimated protein requirements of juvenile fish of a variety of species to be between 30% and 56%. Crude lipid of experimental diets ranged between 16.00% and 19.05%. Apart from satisfying the requirements of a fish for essential fatty acids, dietary lipid acts as a source of energy in diets. Cowey and Sargent (1979) reported that 10%–20% lipid in fish diets gives optimal growth rates without producing excessively fatty carcass. There was a significant variation (*p *≤ .05) in crude protein and lipid of whole body composition of *Heteroclarias* fingerlings‐fed *T. Occidentalis* seed meal diets when compared with the control (Table 3). The reason for this is not far‐fetched from the high protein and lipid contents in *T. Occidentalis* seed meal. Lipids play a major role in energy supply and serve as a vector during intestinal absorption of liposoluble vitamins and carotenoid pigments (Sheridan, 1988). Higher lipid contents were recorded in *Heteroclarias*‐fed *T. occidentalis* seed meal supplemented diets. The main storage sites for lipids in fish are the liver, visceral adipose tissue, or the muscle. High lipid in fish diets when fed *ad libithum*, lead to modifications in body compositions as a percentage of body weight. As the lipid content also increases in carcass, the moisture content decreases, with protein content remaining unchanged (Watanabe, 1982). Guillaume, Kaushik, Bergot, and Metailler (2001) gave a range of 7%–10% as optimal fat incorporation level for some tropical freshwater omnivorous fish species. There was also a significant variation (*p *≤ .05) of ash content in the body of *Heteroclarias* fed the test diets, signifying higher mineral deposition in fish muscle. Ladeji et al. (1995) and Christian (2007) reported that elemental analysis of *T. occidentalis* seed contains higher mineral contents. The assertion by Eyo (2004) that there is reduction in moisture content as lipid deposition increases was also confirmed in this study. Minerals are needed by animals to maintain most of their metabolic processes and to provide material for major structural elements (De Silva & Anderson, 1995).

Growth indices in *Heteroclarias* fed the treatment and control diets revealed significant variations (*p *≤ .05). There was a decline in mean weight gain of fish with increase in the quantity of *T. occidentalis* seed meal in fish diets. This trend was also observed in other parameters such as specific growth rate, protein efficiency ratio, and feed intake. The best FCR was observed in *Heteroclarias*‐fed diet TOSM2 (25% supplementation) level. This implies that the fish would have to consume a feed of 3.39 g to gain 1 g of flesh. Protein efficiency ratio is measured as a quality indicator of amino acid balance in fish diets. This parameter is used to assess protein utilization and turnover whenever there is a partial or total replacement of a protein feedstuff. The best protein intake and feed intake were observed in fish‐fed diet TOSM2. The gradual decline in mean weight gain could be attributed to low feed intake with increase in the quantity of *T. Occidentalis*. Most plant seeds contain 60%–80% phosphorus in the form of phytate (phytic acid), which is not available to the animal (Ravindran, Ravindra, Sivakanasam, & Rajaguru, 1995). The phytic acid also exerts a negative influence on the solubility of proteins and function of pepsin. The phosphate groups of the inositol ring can bind various cations such as calcium, magnesium, iron, and zinc in a fixed complex and thus interfere with their availability. As mentioned earlier, seeds of *T. Occidentalis* used in this experiment was full‐fat, as such, after feed preparation, diets were found to be very oily. Excessive oil reduces acceptability of feed by fish coupled by a drastic reduction in the dissolved oxygen content. These conditions amount to deterioration of water quality as a result of unconsumed feed and cause rancidity of oil in feed when exposed to atmospheric oxygen (Hillestad et al., 2005). Furthermore, low percentage survival was recorded in *Heteroclarias* fingerlings‐fed full‐fat *T. occidentalis* as a result of cannibalism. This behavior depicts that fish were actually hungry and the low feed intake was not sufficient to meet their daily metabolic and physiological functions. On several occasion, head and some skeletal parts of fish were found in water as the flesh would have been consumed by the voracious fishes. At the end of the experiment, the number of *Heteroclarias* remaining in each treatment was 30 fingerlings in TOSM 1 (control), 24 in TOSM 2, 23 in TOSM 3, 27 in TOSM 4, and 21 in TOSM 5.

Water quality determines to a great extent the success or failure of a fish cultural operation and determines the growth, physiology, and health of the animal (Camus, Burrow, Hemstreet, Thure, & Hawke, 1998 and Piper et al., 1982). Water quality parameters measured in this experiment were dissolved oxygen, temperature, pH, nitrate, and ammonia. The dissolved oxygen ranged from 3.50 to 5.75 mg/L and there was a decrease in DO with increase in *T. occidentalis* seed meal. Reduction in DO content could be attributed to the oily nature of experimental diets, leaving a thin oil film on surface water, thereby, preventing diffusion and saturation of atmospheric air. Dissolved oxygen of 5–10 mg/L in culture water favors productivity (Ovie & Adeniji, 2000). Temperature range was between 26.80 and 27. 50°C, and according to Boyd and Lichthoppler (1979), the water temperature range for freshwater fishes is 24.50–29.50°C. The levels of water temperature monitored were within the reported range for hybrid catfish as supported by Abu, Gabriel, Sanni, and Akinrotimi (2009), Nwabueze and Agbogidi (2006), and Oguguah, Nwadukwe, Atama, Chidobem, and Eyo (2011). The pH value of 7.20–7.34 obtained in this study is in conformity with that reported for freshwater fishes (7.40–7.55) by Belarin (1979) and Boyd (1982). Ammonia (0.002–0.003) mg/L and nitrate (0.11–0.13) mg/L levels in culture water were within the ranges recommended by Pronob, Khogen, Sagar, and Bgagabati (2011) for culturing of pond fishes.

## 4 CONCLUSION

This study revealed that partial or total supplementation of *T. Occidentalis* seed meal in the diet of *Heteroclarias* actually led to higher protein, lipid, and ash contents in muscle but had poor performance in their growth. Further processing (defatting) of *T. Occidentalis* seed meal could further unlock and release other potential nutrients. The result of this experiment showed that full‐fat *T. occidentalis* seed meal can replace soya bean meal in the diet of *Heteroclarias* at a minimal level of 25%. Theoretically, the ideal source of protein for an organism is a protein containing same amino acid content and amino acid proportions of the organism itself. Furthermore, if properly processed, *T. occidentalis* (fluted pumpkin) seed meal could replace soybean meal in fish diets to reduce competition between man and animals. From this study, it is recommended that *T. occidentalis* be deffated to remove oil and if not removed, oil should not be included during feed composition in other to reduce its effect on water quality.

## CONFLICT OF INTEREST

None declared.
